# Do rare variant genotypes predict common variant genotypes?

**DOI:** 10.1186/1753-6561-5-S9-S87

**Published:** 2011-11-29

**Authors:** Jack W Kent, Vidya Farook, Harald HH Göring, Thomas D Dyer, Laura Almasy, Ravindranath Duggirala, John Blangero

**Affiliations:** 1 Department of Genetics, Texas Biomedical Research Institute, PO Box 760549, San Antonio, TX 78245-0249, USA

## Abstract

The synthetic association hypothesis proposes that common genetic variants detectable in genome-wide association studies may reflect the net phenotypic effect of multiple rare polymorphisms distributed broadly within the focal gene rather than, as often assumed, the effect of common functional variants in high linkage disequilibrium with the focal marker. In a recent study, Dickson and colleagues demonstrated synthetic association in simulations and in two well-characterized, highly polymorphic human disease genes. The converse of this hypothesis is that rare variant genotypes must be correlated with common variant genotypes often enough to make the phenomenon of synthetic association possible. Here we used the exome genotype data provided for Genetic Analysis Workshop 17 to ask how often, how well, and under what conditions rare variant genotypes predict the genotypes of common variants within the same gene. We found nominal evidence of correlation between rare and common variants in 21-30% of cases examined for unrelated individuals; this rate increased to 38-44% for related individuals, underscoring the segregation that underlies synthetic association.

## Background

A key assumption in genome-wide association studies (GWAS) is that multiple common genetic variants of relatively small effect should make up most of the functional variation responsible for common complex human diseases [1,2]. The genotyping arrays used in GWAS are designed to tag blocks of common variants in high linkage disequilibrium (LD), allowing each array to represent the bulk of the expected functional variation. Despite high expectations, results from GWAS have been modest: Fewer replicable associations have been detected than were initially hoped for, and replicated findings typically account for a small proportion of phenotypic variance, leading to some angst over the missing heritability [3,4]. Dickson et al. [5] pointed out an additional disappointment: resequencing around candidates from GWAS has yielded few identifications of common functional variants in tight LD with the candidate variant. In contrast, molecular dissection of major-gene disorders, such as sickle cell anemia or cystic fibrosis, reveals a pattern of multiple rare causal variants, often spanning several haplotype blocks and each segregating in a relatively small number of pedigrees.

Dickson et al. [5] raised the possibility that association signals obtained from common marker variants might reflect the effects of correlated rare functional variants, a phenomenon they call synthetic association. They demonstrated synthetic association in simulated data and in two human monogenic disorders, sickle cell anemia and familial hearing loss. A striking observation was that a common variant may show indirect association to a phenotype because of the effects of rare variants at considerable physical distance: up to 2.5 Mb, or at least an order of magnitude greater than the expected range of LD, as estimated by, for example, HapMap.

An important aspect of synthetic association, as noted by Dickson et al. [5], is that the observed association between rare and common variants is purely stochastic. The logical converse of the synthetic association hypothesis is that rare variant genotypes must be correlated with common variant genotypes—independently of phenotype—often enough to make synthetic association possible. Here we use exome genotype data from the 1000 Genomes Project [http://www.1000genomes.org] to characterize the extent of correlation between rare and common variants within the same gene.

## Methods

### Data

We used the real exome genotype data from 697 unrelated individuals as provided by the 1000 Genomes Project as well as the simulated genotypes for 697 related individuals from Genetic Analysis Workshop 17 (GAW17) [6]. Because the related individuals were simulated by gene dropping within pedigrees from 202 founders drawn from the 1000 Genomes Project data, these genotypes reflect the effect of within-pedigree allele transmission on real distributions of rare and common polymorphisms (with the caveat that recombination was not permitted within genes). Our work was done with knowledge of the answers to the GAW17 phenotype simulations; however, because our focus was solely on genomic structure, we did not consider whether or not variants were putatively functional in the simulated genetic models for the GAW17 phenotypes.

All analyses were performed in SOLAR [7]. Different investigators have used different criteria for categorizing rarity and commonness. To force the distinction between the two categories, we defined common variants as single-nucleotide polymorphisms (SNPs) with minor allele frequency (MAF) greater than 0.10 and rare variants as SNPs with MAF less than 0.01 with no minor allele homozygotes present. We chose for analysis 686 genes that had at least 10 but not more than 50 variants, at least one of which was common. For each variant, we computed individual genotype scores as the number of copies of the minor allele. For each gene, the genotype score for a randomly chosen common SNP (common variant genotype score, CVGS) was regressed on the unweighted sums of genotype scores for all rare SNPs in the gene (sum of rare variant genotype scores, SRVGS), and the frequency of nominally significant (*P* < 0.05) regressions was recorded. Because our interest in this study is to gain a sense of how often rare alleles appear in coupling with common variant minor alleles, our rather crude SRVGS ignores the possibility that rare variants may act to either increase or decrease a phenotype of interest. In a proper genome-wide association study this would be dealt with by using, for example, a more sophisticated collapsing method [8].

In the family data, the regression models included the random effect of kinship and the fixed effect of the SRVGS. Results were compared for the unrelated individuals and family data sets; to account for the possibility of genotypic correlations introduced by selection, we also classified the results by type of polymorphism (synonymous vs. nonsynonymous changes in coding for amino acids).

Principal components correction for population stratification was performed for one series of tests in unrelated individuals. Principal components were computed in R using the 10,113 synonymous SNPs; individual scores on the first four principal components were used as covariates in the stratification-corrected analyses.

## Results and discussion

### Effects of relatedness and SNP functional type

There was modest evidence of correlation between common and rare variant genotypes in the unrelated individuals, with 21–30% of regression tests showing nominally significant evidence (Table 1). Because ethnic stratification may have a substantial effect on synthetic association [9], we repeated the analyses including principal components correction (see Methods section). Principal components correction reduced the number of nominally significant regressions (19.1% vs. 24.8% without correction) and also reduced both the mean and the standard deviation of the distribution of the regression likelihood ratio test statistics (mean±SD: 2.70±4.83 vs. 3.58±6.90 without correction).

For the families, the proportion of tests reaching nominal significance was substantially larger (38–44%) (Table 2). This result was not unexpected—related individuals will necessarily have more highly correlated genotypes than unrelated individuals—but the result underscores the segregation effects that are the basis of synthetic association (see Conclusions section).

**Table 1:** Significant regressions for unrelated individuals

Rare or common variant type	*N* significant	*N* total	% significant
Nonsynonymous/nonsynonymous	89	298	29.5
Nonsynonymous/synonymous	74	397	21.3
Synonymous/nonsynonymous	77	282	27.3
Synonymous/synonymous	91	330	27.6

The number of nominally significant (*P* < 0.05) regressions of the CVGS and the SRVGS did not differ significantly according to functional type (synonymous vs. nonsynonymous) of rare or common variants.

As noted in the Methods section, we classified regression results according to functional type (synonymous vs. nonsynonymous), expecting that selection might induce correlation of similar functional types. However, we saw no evidence of this in either the unrelated individuals or the family members (Tables 1, 2).

**Table 2:** Significant regressions for family members

Rare or common variant type	*N* significant	*N* total	% significant
Nonsynonymous/nonsynonymous	136	312	43.6
Nonsynonymous/synonymous	137	362	37.8
Synonymous/nonsynonymous	119	271	43.9
Synonymous/synonymous	148	350	42.3

As in unrelated individuals, the number of nominally significant regressions did not differ significantly by variant functional type. However, the percentage of significant regressions was substantially higher for all functional classes than in unrelated individuals.

### Relationship to linkage disequilibrium

**Figure 1:**
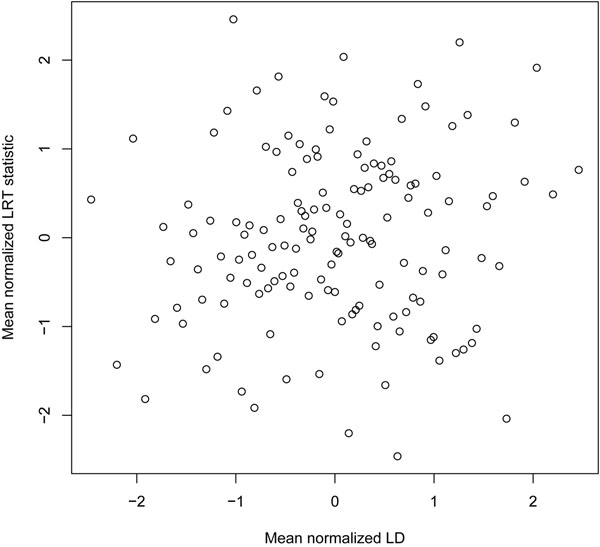
**Comparison of average linkage disequilibrium to results of CVGS and SRVGS regression**. Comparison of normalized mean LD (as |*ρ*|) to likelihood ratio test (LRT) statistics from the regressions for sets of selected common and rare nonsynonymous SNPs (data for unrelated individuals).

Both LD and synthetic association are based on correlations between SNP genotypes, although, as noted, synthetic association is observed to act over much larger genomic distances than LD. In our analyses of nonsynonymous rare and common SNPs in unrelated individuals, we found generally low levels of LD overall (mean±SD within-set LD = 0.049±0.060, range 0.007–0.383). There was little evidence of association between the average LD in the set and the regression results (Figure 1), although the pairwise LD calculations are presumably imprecise because of the distribution of the rare alleles.

### Specific examples

The comparison between unrelated individuals and family members is complicated by the substantial number of rare variants that became monomorphic in the families as a result of founder effects; for direct comparisons of common variants, the mean (SD) number of rare variants was 10.28 (6.73) in the unrelated individuals and 4.58 (5.42) in the families. It is well known that familial segregation may stochastically amplify the copy number of rare variants (although, of course, it may also force the major allele to fixation). This may be reflected in the similarity in distribution of the SRVGS per common variant: mean (SD) for SRVGS = 0.0192 (0.013) in unrelated individuals and 0.011 (0.013) in families despite the difference in the number of rare variants.

An interesting alternative explanation for the generally greater degree of genotypic correlation in the families is that the process of segregation induces gametic phase disequilibrium between rare and common variants. Although direct evidence of this is limited in our data, Table 3 presents four cases in which the set of rare variants was identical in both the unrelated individuals and the families; in each case, the test statistic for regression of the CVGS on the SRVGS is greater in the families, supporting the hypothesis of genotypic correlation resulting from familial segregation.

**Table 3:** Direct comparison of regression results in unrelated individuals and families

Locus	Common SNP	Number of rare SNPs	*β*, unrelated individuals	LRT, unrelated individuals	*β*, families	LRT, families
*MKI67*	C10S6822	6	0.11	3.43	0.15	3.88
*WFDC3*	C20S1341	4	−0.08	0.58	−0.18	7.67
*LASS4*	C19S921	2	−0.01	0.00	0.02	0.01
*ZNF571*	C19S3534	1	1.16	2.61	1.20	3.01

When the set of rare variants is the same in regression tests for both the unrelated individuals and the family members, evidence for correlation between the CVGS and the SRVGS is greater in the families. *β* = regression slope; LRT = likelihood ratio test statistic (distributed as a chi-square distribution with 1 degree of freedom).

## Conclusions

The phenomenon of synthetic association—association of common variants with disease because of accidental genotypic correlations between common marker variants and functional rare variants—implies that such correlations should occur fairly often. We have used the exome data provided by GAW17 to characterize the extent of such correlation without reference to any phenotype (that is, our interest was not in synthetic association per se but rather in its genomic origin). In a random draw of common variants, we found evidence of nominally significant prediction of the CVGS by the SRVGS in about 20–30% of cases in unrelated individuals and in about 40% of cases in families. Thus stochastic correlation between rare and common variant genotypes appears to occur frequently enough that synthetic association need not be a rare occurrence. We found no evidence that this correlation is due to selection, at least by comparison of synonymous and nonsynonymous coding SNPs.

Although the increase in evidence for genotypic correlation in families may be due to several factors, we find some evidence to support a role for gametic phase disequilibrium induced by familial segregation. Interestingly, Sun et al. [9] found a similar increase in correlation by stratifying the unrelated individuals by ethnic background, suggesting a similar influence of ancestry and founder effect. Therefore synthetic association may result from synthetic haplotypes stochastically generated by gametic phase disequilibrium in population strata, whether these are ancestral or familial. If this suggestion is supported by future investigations, it reinforces the utility, if not the necessity, of taking relatedness into account in the search for genetic variants that contribute to common complex diseases.

## Competing interests

The authors declare that there are no competing interests.

## Authors’ contributions

JB and RD conceived of the study. JWK designed the study, performed the analyses, and co-wrote the manuscript. VF co-wrote the manuscript. HHHG, LA, and TDD provided helpful commentary and technical advice. All authors read and approved the final manuscript.
